# The Relationship Between Attitude Toward Science and Academic Achievement in Science: A Three-Level Meta-Analysis

**DOI:** 10.3389/fpsyg.2021.784068

**Published:** 2021-12-16

**Authors:** Peipei Mao, Zhihui Cai, Jinbo He, Xinjie Chen, Xitao Fan

**Affiliations:** ^1^School of Psychology, Central China Normal University, Wuhan, China; ^2^School of Humanities and Social Science, The Chinese University of Hong Kong, Shenzhen, China; ^3^Graduate School of Education, Stanford University, Stanford, CA, United States; ^4^Faculty of Education, The Chinese University of Hong Kong, Hong Kong, Hong Kong SAR, China

**Keywords:** attitude toward science, science achievement, meta-analysis, moderator analysis, academic achievement in science

## Abstract

Science education is attracting increasing attention and many researchers focus on the issue about the attitude-achievement relationship in science, but there is still no consistent conclusion. By using a three-level meta-analytic approach, the aim of the current study was to investigate the relationship between attitude toward science and academic achievement in learning science among primary and secondary school students, and to explore if some study characteristics could have contributed to the inconsistent findings with regard to this relationship as observed in the research literature. A total of 37 studies with 132 effect sizes involving a total of 1,042,537 participants were identified. The meta-analytic results revealed that there was an overall positive and moderate relationship between attitude toward science and learning achievement in science (*r* = 0.248, *p* < 0.001). The results further found that this association was moderated by the type of attitude and larger effect sizes were shown in self-efficacy than in interest, societal relevance of attitude toward science, and mixed attitude. Moreover, the effect sizes of studies with unstandardized measure to assess science achievement were larger than those with standardized measure. Possible explanations for these findings and its implications for future research directions were also discussed in this review.

## Introduction

Science education is an important subject area of study for students, as it is closely tied to a society's economic development. In addition to students' achievement in learning science, affective outcomes related to science learning are also of concern for educators. Over the years, science education educators and researchers have been interested in understanding the relationship between students' attitude toward science and their achievement in learning science (e.g., Abu-Hilal et al., 2014; Darmawan, 2020). One of the purposes of science education is to develop a positive attitude toward science and to enhance the interest of young people in pursuing scientific careers (Tai et al., 2006; Azizoglu and Çetin, 2009). In recent years, the decline of students' favorable attitude toward science and the falling number of students choosing to pursue the study of science have become a matter of considerable societal concern and debate in some regions across the world (e.g., Kennedy et al., 2014; Potvin and Hasni, 2014a; Cheng and Wan, 2016). For the economic development of a society, lack of positive attitude toward science and the low interest among the young to pursue science careers pose serious threats to economic prosperity (Osborne et al., 2003; Kennedy et al., 2016). Therefore, it is necessary to understand how the attitude toward science and achievement in learning science are related among young learners, such that we may develop a better understanding about how this relationship may affect students' choice of subject areas for learning.

Over the years, there have been many empirical studies concerning the science attitude-achievement relationship. The research literature in this area, however, has not provided consistent findings. To address this issue, some literature reviews on attitude toward science were done (Aiken and Aiken, 1969; Gardner, 1975; Osborne et al., 2003), and a meta-analysis of the research literature on the attitude-achievement relationship in science was conducted several decades ago (Willson, 1983), which reported some basic descriptive statistics (e.g., mean or median of correlation coefficients), and issues (e.g., weighted analysis by study sample size) were not considered. It has been about four decades since the last quantitative synthesis of research on this issue, and the relevance and validity of the previous findings are very much in question. Therefore, this study was designed for the purpose of providing an up-to-date quantitative synthesis of the research literature in recent decades on the relationship between attitude toward science and achievement in science learning by using the most current meta-analytic methods. More specifically, the three-level meta-analytic approach (Assink and Wibbelink, 2016) is used, and this meta-analytic model adequately addresses multiple technical issues, including dependent effect sizes from the same study.

### Considerations for Attitude Toward Science

“Attitude could be considered as people's global evaluations of any object, such as oneself, other people, possessions, issues, abstract concepts, and so forth” (Petty et al., 2003). In the area of research for studying attitude, the biggest stumbling block is often the lack of clarity about the concept under investigation. Klopfer (1971) made a notable contribution by proposing six dimensions regarding affective behaviors in science, namely, attitude toward scientists, scientific enquiry, science learning, science-related activities, science careers, and the adoption of “scientific attitudes.” More clarity gradually emerged across studies, as the studies became clearer in what components or measures were used for attitudes toward science (Schibeci, 1983; Breakwell and Beardsell, 1992; Woolnough, 1994; Koballa, 1995). According to Osborne et al. (2003), attitude toward science can be defined as “feelings, beliefs and values held about the enterprise of school science, and the impact of the science on society.” However, such definitions either consist of a single unitary construct, or consist of multiple sub-constructs. Reid (2006), on the other hand, holds that attitude can be divided into three components: cognitive, affective, and behavioral. In addition, Potvin and Hasni (2014b) argue that attitude contains a wide range of subconstructs, such as enjoyment, motivation, self-efficacy, and career aspirations. Thus, it is obvious that the conceptual frameworks of attitude are diverse.

One possible reason for the inconsistent findings across individual studies about the relationship between science attitudes and science achievement was that different studies might have operationalized the construct of attitude differently. Based on our review of relevant research literature in the area of attitude toward science, in this study, we drew on Savelsbergh et al. (2016) fine grained framework of attitude constructs and grouped different operationalization of attitude toward science across the studies into four categories: interest, self-efficacy, societal relevance of attitude toward science and mixed attitude. The interest aspect of science is represented by the emotions and feelings about learning science (e.g., Zhang and Tang, 2017). The self-efficacy aspect of science attitude involves students' beliefs in their own abilities to achieve good grades in science-related subject courses, to be competent in relevant science careers, and to undertake tasks in science successfully (e.g., Larson et al., 2014). The societal relevance of attitude toward science is represented as the perceptions and judgement about the value, usefulness, social implications of science (e.g., Dowey, 2013). Finally, some studies either focused on students' general science attitude, or did not provide clear description or operationalization of the “attitudes” as measured in the studies, and we classified such cases as “mixed” (e.g., Oluwatelure, 2015).

### Relationship Between Attitude Toward Science and Achievement in Learning Science

Over the past decades, after the last synthesis on the students' attitude -achievement relationships (Willson, 1983), there has been a growing interest on this issue, and the studies in the recent two decades continue to provide inconsistent findings. On the one hand, many studies showed that students' attitude toward science and their science achievement correlated positively and moderately (Nolen, 2003; Mungin, 2012; Ng et al., 2012; Hacieminoglu, 2016; Chi et al., 2017; Wang and Liou, 2017; Zheng et al., 2019). For instance, based on the data from Program for International Student Assessment (Pisa et al., 2017), the study conducted by Chi et al. (2017) pointed out that students' interest, enjoyment, and the perceptions of general value in science were positively correlated with scientific competencies. Similarly, in another study with 537,170 15-year-old students, Zheng et al. (2019) stated that students' interest in science was positively associated with their science achievement. Meanwhile, based on the Chinese sample of Trends in International Mathematics and Science Study (Martin et al., 2012), Wang and Liou (2017) revealed that students' perception about the intrinsic value and utility value of science had a significant positive effect on their science learning performance. Furthermore, some research studies showed a strong relationship between attitude and achievement (Mattern and Schau, 2002; Else-Quest et al., 2013; Oluwatelure, 2015). For example, in the study by Oluwatelure (2015), a significant and strong positive correlation between science attitude and science achievement (*r* = 0.612) was shown. Likewise, Rennie and Punch (1991) documented that students' beliefs in their performance was closely related to science achievement (*r* = 0.66).

On the other hand, however, there were other studies showing that the relationship between students' attitude toward science and their science achievement was either quite weak, statistically non-significant, or even negative (Rennie and Punch, 1991; Gardner, 1995; Brooks, 2011). For example, Brooks (2011) revealed that the enjoyment of science lessons, leisure interest in science activities, social implications and career in science of students were negatively associated with their science achievement. Moreover, there were also some studies that yielded contradictory results (Napier and Riley, 1985; Diggs, 1997; Salmi et al., 2016). For instance, based on a sample of sixth grade students from Finland, Estonia, Latvia and Belgium, Salmi et al. (2016) reported that the correlation between students' societal attitude (value of science in society) and performance was positive (*r* = 0.11), but the relationship between students' engineering attitude (interest in computer design) and performance was negative (*r* = −0.11).

### Study Characteristics as Possible Factors for Inconsistent Findings

As discussed above, empirical studies have shown inconsistent findings with regard to the relationship between attitude toward science and achievement in science learning. As discussed extensively in meta-analytic research literature, some study features may have contributed to the inconsistent findings as shown in the research literature (so-called “moderators” in meta-analytic studies). In this section, we consider some possibilities in this regard.

#### Publication Type

Publication type is a common moderator variable in meta-analysis that captures different types of research publications such as journal article, conference paper, or dissertation. In general, journal articles and some conference papers are peer-reviewed, whereas dissertations are not. Given the belief that studies with statistically significant findings are more likely to be published than those with statistically non-significant findings (i.e., file drawer problem, or publication bias; Rosenthal, 1979), inconsistent findings across studies could be due to different publication types. Thus, publication type was examined as a potential moderator variable in this meta-analytic study. The studies were coded as either “journal article” or “dissertation” in this meta-analysis.

#### Grade

Relevant studies in this area involved students at different grade levels. Previous primary studies on science attitude suggested that the relationship between attitude toward science and science achievement could vary across grade levels. For example, Liou and Liu (2015) noted that the correlation between students' self-concept and science scores, and that between intrinsic interest and science scores, were stronger for the eighth grade students than for the fourth grade students, based on the TIMSS 2011 Taiwanese data. Similarly, Liou et al. (2021) also suggested that this association was stronger for junior middle school students than for elementary school students. With such a consideration for grade level as a possible factor for inconsistent findings in the literature, in this study, we would examine the potential moderating effect of grade level on the relationship between science attitude and learning achievement in science, and we coded the grade levels of the studies as having elementary school students for grades 1–6, middle school students for grades 7–9, high school students for grades 10–12, and others (mixed covering more than one grade level).

#### Geographical Region

Studies about science attitude and science learning involved participants from different geographical regions (e.g., USA, Turkey, and China). In addition, previous research indicated that there were regional differences in science achievement (Martin et al., 2012; Bati et al., 2019). For example, research on international students' science achievement showed that there were differences across countries in TIMSS 2011 at the fourth grade, with some countries (e.g., Finland, Korea, Singapore) showing considerably higher level of achievement than some others (Martin et al., 2012). Furthermore, Bati et al. (2019) investigated the degree to which affective characteristics could predict students' science performance based on the results from PISA 2015, and suggested that science self-efficacy of students could significantly predict science achievement in samples from multiple countries. Thus, the strength of the association between science attitude and science learning achievement could vary across countries/regions. With this consideration, in our meta-analysis, geographical region where a study was conducted would be coded as a potential factor for the inconsistencies of findings across the studies, and the geographical regions of the studies were coded into one of two regions based on the relevant information in the included primary studies: Eastern countries (e.g., China, Singapore) and Western countries (e.g., USA, Italian).

#### Type of Attitude

As discussed above, attitude is a complex construct, and different operationalization and measurement of this construct in primary studies could have led to inconsistent findings across the studies in this area. For example, Else-Quest et al. (2013) examined the link between attitude toward science and science achievement, and suggested that the self-concept of ability in science showed stronger link with academic outcomes than science value did. Similarly, Chang and Cheng (2008) reported that students' self-confidence was a better predictor for achievement than their interest in science in a student sample from Taiwan. In this study, type of attitude used in a study was treated as a potential factor for the inconsistent findings about the relationship between students' attitude toward science and their science achievement in different studies. More specifically, as discussed earlier, we coded “attitude” in the primary studies into one of the four categories: interest, self-efficacy, societal relevance of attitude toward science and mixed (usually not sufficiently clear to be classified into any of the three categories before).

#### Measures of Achievement

Studies in this area used different measurements for academic achievement in science. Measures used in the studies for science achievement generally fell into one of two categories: standardized measures/tests and unstandardized assessments. Standardized measures/tests, such as those used in large-scale projects like TIMSS and PISA, are believed to have high levels of validity and reliability as a result of development efforts for these measures/tests (e.g., Hamilton, 1982; Oliver and Simpson, 1988; Cohen and Chang, 2020). Unstandardized assessments may have various forms, such as school grades in science and science-related GPA, and these are typically created by teachers or researchers (e.g., Schibeci and Riley, 1986; Freedman, 1997; Dowey, 2013). These two types of measures of science achievement could have some differences. Wiberg and Rolfsman (2019) in their study involving both the science achievement measure in TIMSS and measure of school science achievement discussed that the association between the two kinds of measures was moderate, and that the contents of TIMSS measure of science achievement were not always in accordance with the school system. Furthermore, as Jansen et al. (2014) demonstrated, students' academic self-concept in science showed more pronounced relationship with their final science grades than with their scores on standardized tests. With such considerations, in our study, measures of science achievement was treated as a potential moderator, and the measures used in the studies included in this meta-analysis were grouped into either “standardized” or “unstandardized” categories.

#### Publication Year

Several decades ago, Willson (1983) showed that the magnitude of the relationship between attitude toward science and science achievement did not vary significantly over time. However, with more emphasis on science education in recent decades, and with the reform efforts in science education curriculum and instruction, students' attitudes toward science and science achievement may change over time. For example, based on the data from TIMSS assessments of the fourth grade students in multiple countries, Martin et al. (2012) discussed that students' performance in most countries increased during the period of 1995–2011 period. Considering the possible changes in both student attitude toward science and learning achievement in science in recent decades, we included the final publication year of a research publication (either a journal article or a dissertation) as a potential moderator in the present study.

### Study Aims

This meta-analysis was planned to conduct a systematic quantitative synthesis of the empirical studies that examined the relationship between attitude toward science and learning achievement in science. This quantitative synthesis would provide an accurate, reliable, and valid summary of the research findings on this issue, and would allow us to understand if some features of the individual studies might have contributed to the inconsistent findings across the individual studies. More specifically, this study was designed to address the following two major questions:

1. What is the magnitude of the general relationship between students' attitude toward science and their science achievement?
2. Does the relationship between attitude toward science and achievement in science vary due to some study features of the individual studies, such as publication type, grade level, geographical region of the sample, type of attitude, measures of achievement, and publication year?

## Methods

### Literature Search for Primary Studies

To obtain the studies to be included in this meta-analysis, ERIC, PsycINFO, SAGE, Taylor & Francis Online, and ProQuest Dissertations & Theses Global were used to identify studies examining the relationship between attitude toward science and academic achievement in science involving students from elementary school to high school. Key words used were: science, attitude, anxiety, interest, usefulness, value, self-efficacy, self-concept, enjoyment, achievement, and performance. Our search was conducted by using the key words either singly or in different combinations. The search covered the literatures up to October 2020. Google Scholar was also used in the follow-up search to identify additional studies that were not contained in the above databases. Reference sections from the included articles and several related review articles about the relationship between attitude toward science and science achievement were also examined to find additional research articles.

### Inclusion Criteria

To be included in this meta-analysis, studies must meet the following criteria:

1. A study must be published or available in English;
2. A study must examine science attitude and science achievement simultaneously, and had operational definition of attitude toward science;
3. A study must report the zero-order correlation between science attitude and academic outcome in science, or reported quantitative data in sufficient detail to allow us to obtain this relationship as an effect size (e.g., *t*-ratio, *F*-ratio, etc.). We contacted the authors that did not report these correlations in their articles to request this information, and studies with no response were excluded.
4. A study should not include any experimental interventions on either attitude or achievement, or on both, because such intervention might have changed the relationship between the two variables (e.g., Aguilera and Perales-Palacios, 2020b; Sahin and Yilmaz, 2020).
5. Samples of the study must be students from elementary, middle and high schools; college students or other groups were excluded in the present study.

### Selection Procedure

**Figure 1:**
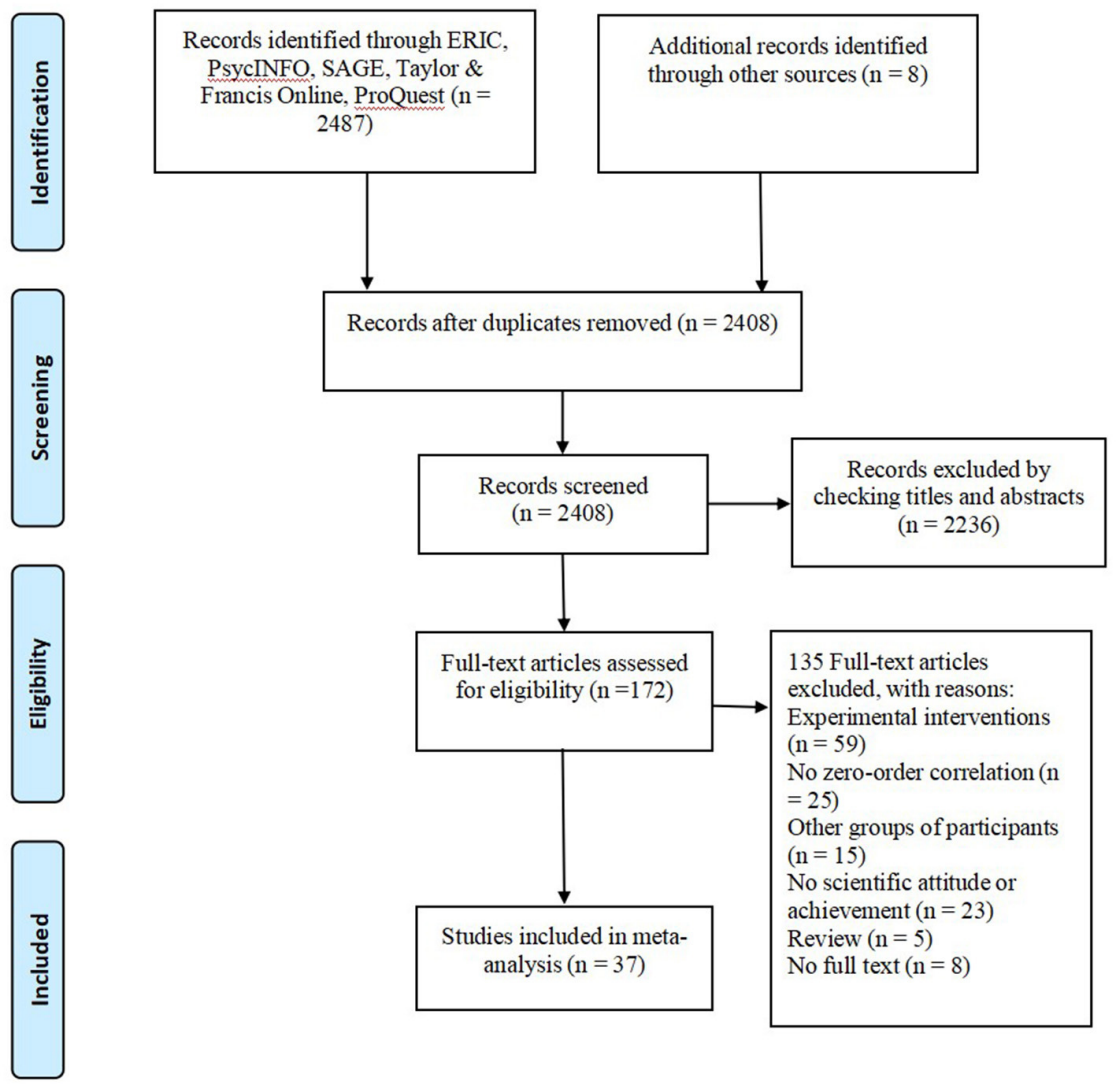
PRISMA flow chart of the selection of studies for the meta-analysis.

The PRISMA flow chart of the selection process was presented in Figure 1. Initially, we obtained 2,408 studies after removing duplicates. Two authors read the titles and abstracts of all articles, and 172 full-text articles were obtained for possible inclusion. Then, they carefully screened these articles based on the aforementioned selection criteria and found that 135 studies did not meet the inclusion criteria. In the end, 37 primary studies were included in this meta-analysis, and these studies were published from 1982 to 2020.

### Coding of Study Features

As detailed previously, to understand what might have contributed to the inconsistent findings across the studies about the relationship between students' science attitude and their academic achievement in science, the following study features were coded in this study: (a) publication year (year as a continuous variable); (b) publication type (published journal article or unpublished dissertation); (c) grade (four levels: elementary school students, middle school students, high school students, and others); (d) geographical region (two categories: Eastern countries and Western countries); (e) type of attitude (four categories: interest, self-efficacy, societal relevance, and mixed); (f) measures of achievement (two categories: standardized and unstandardized). In addition, the first and the second author coded 10% of the randomly selected articles and all disagreements were solved after reading the articles and discussion with the research team. Then, all primary studies were coded independently by two authors with high inter-rater reliability. The intra-class correlation coefficients of each moderator variable were respectively: publication year (ICC = 0.94), publication type (ICC = 1), grade level (ICC = 0.97), geographical region (ICC = 1), type of attitude (ICC = 0.89), and measures of achievement (ICC = 0.86).

### Data Analysis Strategy

The zero-order correlation coefficients *r* between attitude toward science and academic achievement in science from the primary studies were treated as effect sizes in the present meta-analysis. Before conducting the meta-analysis, all correlation coefficients were converted to Fisher's *z*-scores, because the sampling distribution of *r* is skewed (Card, 2012). The Fisher's *z*-scores were transformed back into *r* after performing the meta-analysis. It was important to note that most of the studies included in our meta-analysis reported multiple relevant effect sizes, since correlation coefficients *r* between different dimensions of attitude toward science and different measures of achievement in science could be extracted from the same study. However, the traditional meta-analytic approach assumes that the observed effect sizes should be independent of each other, which is not the case here. Therefore, for the situation of non-independent effect sizes, i.e., effect sizes nested under study, a three-level meta-analysis approach was applied to deal with the dependency of effect sizes in the current study (Assink and Wibbelink, 2016). The models for three-level random-effect meta-analysis are expressed:


$$\begin{array}{lll} \text{Level }1\text{ model}:\;{\text{y}}_{\text{ij}} & = & \lambda_{ij}+e_{ij}\\ \text{Level }2\text{ model}:\;{\text{λ}}_{\text{ij}} & = & \kappa_{j}+\mu_{\left(2\;\right)ij}\\ \text{Level }3\text{ model}:\;{\text{κ}}_{\text{j}} & = & \beta_{0}+\mu_{\left(3\;\right)j} \end{array}$$

The *y*_*ij*_ is the *i*th effect size in the *j*th study, λ_*ij*_ is the “true” effect size, Var(*e*_*ij*_) is the known sampling variance in the *i*th effect size in the *j*th study, κ_*j*_ is the average effect in the *j*th study, β_0_ is the average population effect, and Var$\left(\mu_{\left(2\right)ij}\right)=\tau_{\left(2\right)}^{2}$ and Var$\left(\mu_{\left(3\right)j}\right)=\tau_{\left(3\right)}^{2}$ are the study-specific level 2 and level 3 variance, respectively (Cheung, 2014). In the three-level random effects model, three sources of variances were distributed: sampling variance of the observed effect sizes as level 1; variance within the same study as level 2 $\left(\tau_{\left(2\right)}^{2}\right)$; variance between studies as level 3 $\left(\tau_{\left(3\right)}^{2}\right)\;$(Cheung, 2014). The maximum likelihood estimation method is used to compute the parameter estimates, including *Q* statistic (i.e., the homogeneity of model estimates), and *I*^2^ statistic (i.e., the proportion of the distribution of the total variance over level 1, level 2, and level 3). All analyses were performed by using Viechtbauer, 2010 *metafor* package in R version 3.5.1.

### Publication Bias

Publication bias should be taken into account in conducting meta-analysis. Generally, studies with statistically significant results could be more likely to be published, thus included in a meta-analysis, than those with statistically non-significant results, and this was referred to as the “file-drawer problem” (Rosenthal, 1979). In our meta-analysis, first, we used a funnel plot to assess the presence or absence of publication bias. If the funnel plot was symmetrically distributed, the absence of publication bias was supported (Borenstein et al., 2009). Furthermore, a combined Tandem Procedure was used in publication analyses (Ferguson and Brannick, 2012). Rosenthal's “fail-safe N” method, Egger's regression test, and Begg's correlation test were conducted to assess the potential publication bias. The *p*-values in these tests were >0.05, indicating that there is no enough evidence to suggest publication bias was presented.

## Results

### Characteristics of Included Studies

The present meta-analysis included 37 primary studies containing 48 independent samples and 132 effect sizes, and these studies were published from 1982 to 2020 (see Appendix). More specifically, the number of effect sizes related to the moderator variables varied: Publication year (132 effect sizes); Publication type: *journal* (114 effect sizes), *dissertation* (18 effect sizes); Grade: *elementary school students* (13 effect sizes), *middle school students* (79 effect sizes), *high school students* (29 effect sizes), *others* (9 effect sizes); Geographical region: *Eastern* (42 effect sizes), *Western* (86 effect sizes); Type of attitude: *interest* (43 effect sizes), *self-efficacy* (43 effect sizes), *societal relevance* (30 effect sizes), *mixed* (16 effect sizes); Measures of achievement: *standardized* (70 effect sizes), *unstandardized* (62 effect sizes). In addition, the 37 included studies involved a cumulative total of 1,042,537 participants, with sample size for individual studies ranging from 21 to 537,170.

### Overall Analysis

The overall analysis results of the association between attitude toward science and achievement in science are shown in Table 1. A statistically significant overall weighted mean correlation, *r* = 0.248 (*p* < 0.001), was obtained. It indicated that the relationship between attitude toward science and achievement in science of students was positive and moderate. The *Q* statistic was statistically significant [*Q*_(131)_ = 4111.714, *p* < 0.001], suggesting that the effect sizes across the studies were heterogeneous. Moreover, the results of the likelihood-ratio tests revealed that the variances within-studies (*estimate* = 0.011, *p* < 0.001) and variances between studies (*estimate* = 0.013, *p* < 0.001) were significant. In terms of the total effect size variance, the level 1, level 2 and level 3 accounted for 1.928, 44.617, and 53.456%, respectively. Taken together, moderator analyses are warranted to further explore how study features might have contributed to the inconsistencies of the effect sizes across the studies.

**Table 1:** Results for the overall analysis of the relation between attitude toward science and science achievement.

	**No. studies**	**No. ES**	**Mean *z* (*SE*)**	**95% CI**	***t*-value (sig)**	**Mean *r***	**% var. at level 1**	**Level 2 variance**	**% var. at level 2**	**Level 3 variance**	**% var. at level 3**
Overall association	37	132	0.253 (0.021)	(0.213, 0.294)	12.349***	0.248	1.928	0.011***	44.617	0.013***	53.456

*No. Studies, number of studies; No. ES, number of effect sizes; Mean z, Mean effect size (Fisher's z); SE, standard error; CI, confidence interval; sig, significance; Mean r, Mean effect size (r); Var., variance; Level 1 variance, sampling variance of observed effect sizes; Level 2 variance, variance between effect sizes extracted from the same study; Level 3 variance, variance between studies.*

***p < 0.001.

### Publication Bias

**Figure 2:**
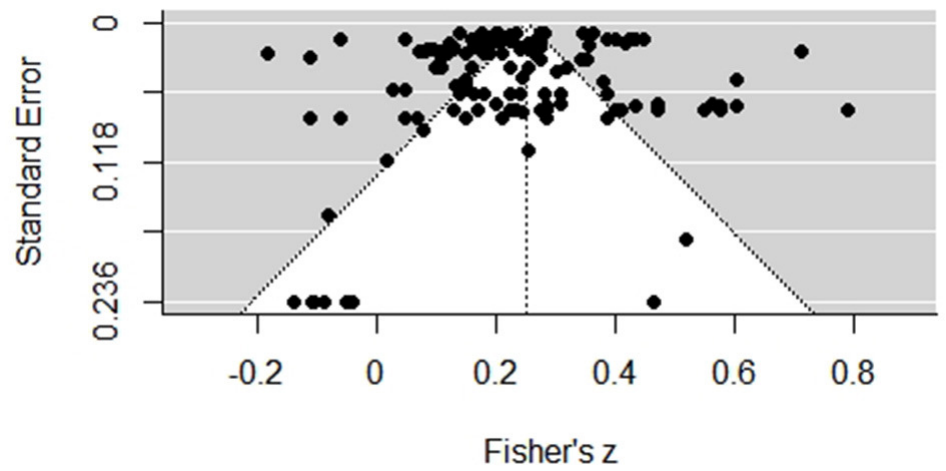
Funnel plot for effect sizes.

To evaluate the potential publication bias, both funnel plot, the multilevel extension of Egger's regression test, Begg's correlation test, and “fail-safe N” method, as proposed by Fernández-Castilla et al. (2019), were used in the current three-level meta-analysis. As shown in Figure 2, the funnel plot is symmetrically distributed, suggesting no evidence of publication bias subjectively. Moreover, the statistically non-significant results of Egger's regression test (*p* = 0.246) and Begg's correlation test (*p* = 0.595) also does not show enough evidence to justify the presence of a publication bias. In addition, the fail-safe *N* was calculated to be 518503, which is much larger than the criteria that 5*k* + 10 = 5 × 132 + 10 = 670. Overall, all of the assessment results indicated the absence of publication bias in this study.

### Moderator Analysis

The results of the moderator analyses on the association between attitude toward science and science performance are presented in Table 2.

**Table 2:** Results for the moderator analysis of the relation between attitude toward science and science achievement.

**Moderator variables**	**No. studies**	**No. ES**	**Mean *z* (*SE*)**	**95% CI**	***t*-value (sig)**	**Mean *r***	***F* (df_**1**_, df_**2**_)**	**Level 2 variance**	**Level 3 variance**
**Publication type**	37	132					*F*_(1, 130)_ = 3.983*	0.011	0.012
Journal	32	114	0.266 (0.021)	(0.224, 0.307)	12.610***	0.260			
Dissertation	5	18	0.123 (0.068)	(−0.013, 0.258)	1.791	0.122			
**Grade**	35	130					*F*_(3, 126)_ = 0.602	0.011	0.014
Elementary school	6	13	0.219 (0.058)	(0.104, 0.334)	3.779***	0.216			
Middle school	24	79	0.273 (0.028)	(0.218, 0.329)	9.780***	0.266			
High school	7	29	0.256 (0.048)	(0.161, 0.352)	5.297***	0.251			
Others	3	9	0.176 (0.080)	(0.018, 0.333)	2.207*	0.174			
**Geographical region**	33	128					*F*_(1, 126)_ = 0.051	0.010	0.011
Eastern	12	42	0.251 (0.032)	(0.187, 0.314)	7.837***	0.246			
Western	21	86	0.241 (0.026)	(0.189, 0.293)	9.204***	0.236			
**Type of attitude**	37	132					*F*_(3, 128)_ = 11.560***	0.009	0.009
Interest	20	43	0.208 (0.024)	(0.160, 0.256)	8.541***	0.205			
Self-efficacy	14	43	0.325 (0.025)	(0.276, 0.374)	13.151***	0.314			
Societal relevance	13	30	0.169 (0.028)	(0.113, 0.224)	6.019***	0.167			
Mixed attitude	14	16	0.294 (0.035)	(0.226, 0.363)	8.487***	0.286			
**Measures of achievement**	37	132					*F*_(1, 130)_ = 7.001**	0.010	0.015
Standardized	22	70	0.214 (0.026)	(0.163, 0.265)	8.307***	0.211			
Unstandardized	16	62	0.314 (0.031)	(0.252, 0.376)	10.083***	0.304			
**Publication year**	37	132	0.003 (0.002)	(−0.001, 0.006)	1.443	0.003	*F*_(1, 130)_ = 2.083	0.011	0.013

*No. Studies, number of studies; No. ES, number of effect sizes; Mean z, Mean effect size (Fisher's z); SE, standard error; CI, confidence interval; sig, significance; Mean r, Mean effect size (r); F (df1, df2), omnibus test; Level 2 variance, variance between effect sizes extracted from the same study; Level 3 variance, variance between studies.*

*p < 0.05; **p < 0.01; ***p < 0.001.

#### Publication Type

There were a total of 132 effect sizes in this study, with 114 from journal articles and 18 from dissertations. We found a statistically significant amount of explained effect-size heterogeneity for the publication type moderator [*F*_(1, 130)_ = 3.983, *p* < 0.05] and the effect sizes of journal articles (*r* = 0.260) appeared to be larger than those from dissertations (*r* = 0.122).

#### Grade

No significant differences were observed when considering the moderator effect of grade [*F*_(3, 126)_ = 0.602, *p* = 0.615], suggesting that attitude toward science among elementary school students, middle school students, high school students and others was all positively related to their science achievement with some consistency (*r* = 0.216; *r* = 0.266; *r* = 0.251; *r* = 0.174, respectively).

#### Geographical Region

In line with the grade, the geographical region did not explain a statistically significant amount of effect-size heterogeneity [*F*_(1, 126)_ = 0.051, *p* = 0.822]. Studies with samples from Eastern countries had the mean effect size of 0.246, and 0.236 for Western countries.

#### Type of Attitude

We found a significant moderating effect of attitude types [*F*_(3, 128)_ = 11.560, *p* < 0.001]. More specifically, the strength of the correlation between science performance and self-efficacy about science (*r* = 0.314) was stronger than that of interest (*r* = 0.205), societal relevance of attitude toward science (*r* = 0.167), and mixed attitude (*r* = 0.286).

#### Measures of Achievement

There are various instruments to measure achievement. We mainly divided the instrument types into two groups: standardized test and unstandardized assessment. The effect of the moderator was significant [*F*_(1, 130)_ = 7.001, *p* < 0.01]. The effect sizes of studies with unstandardized measure were larger (*r* = 0.304) than those with standardized measure (*r* = 0.211).

#### Publication Year

Studies included in our meta-analysis were published from 1982 to 2020 and there was a long time span. Our analysis showed that the association between attitude toward science and academic achievement in science did not appear to have changed with the time, with the slop of this regression model being statistically non-significant (β = 0.003, *p* = 0.151), indicating that the magnitude of effect sizes remained stable over the time period.

## Discussion

This meta-analysis systematically synthesized the findings of the studies from 1982 to 2020 on the relationship between attitude toward science and academic achievement in science, and aimed to estimate the magnitude of overall association between the two variables. In addition, the study explored if some study features (publication type, grade level, geographical region, type of attitude, measures of achievement, and publication year) could have contributed to some observed inconsistent findings about this relationship across the studies.

Our investigation showed that, across the studies conducted over the past several decades, the overall correlation between attitude toward science and academic achievement in science was 0.248, a moderate positive association. Similar findings had been found in previous empirical studies (e.g., Acar et al., 2015; Li et al., 2020; Liou et al., 2021). This relationship suggests that enhancing students' positive attitude toward science could be conductive to students' learning in science. The quantitative literature synthesis by Savelsbergh et al. (2016) showed that some context-based teaching approaches (e.g., inquiry-based learning, technology-based learning environments, collaborative learning, and extracurricular activities) could have significant positive influence on students' overall attitude toward science. The meta-analytic study by Aguilera and Perales-Palacios (2020a) showed similar findings that some teaching methods/approaches (e.g., cooperative learning, project-based instruction, context-based instruction, and technology-multimedia materials) could lead to positive change in students' attitude toward science. In the future, these learning strategies and other emerging teaching methods, such as flipped learning, game-based learning, etc., can be used in science education to assist students to develop more positive attitude toward science.

In this study, a set of study features (i.e., publication type, grade, geographical region, type of attitude, measures of achievement, and publication year) were examined for their possible contributions to the inconsistent findings across individual studies. Finally, publication type, the type of attitude, and measures of achievement were shown to have moderating effects on the effect sizes of the studies. These findings, or lack thereof, were discussed below.

### Publication Type

Our analysis found that the average effect size from journal articles was larger than that from dissertations. This may be due to the small number of dissertations (only five articles) in the included studies. However, based on the fact that our previous results did not show enough evidence to justify the presence of publication bias, this finding should be interpreted with caution.

### Grade

Our synthesis showed that there was no significant difference among students at different grade levels (e.g., elementary, middle, high school), and science attitude had a moderate positive relationship with science learning in these grade levels. An issue that students' s attitudes toward science might decline from elementary to middle school is received with concern (Potvin and Hasni, 2014a). This finding of our study indicated that educators should pay attention to students' attitudes toward science at the elementary-school level and make the appropriate intervention.

### Region

The analysis showed no significant divergence across the studies conducted in different regions (Eastern countries and Western countries). This result was in agreement with some other research showed that the general relationship between attitude toward science and knowledge of scientific facts varied little across different countries (Allum et al., 2008). Research also suggested that this lack of difference was also observed across countries within a region (e.g., Malaysia vs. Singapore, in Ng et al., 2012). On a somewhat different issue, however, Lam and Lau (2014) suggested that students of Asian countries might have high performance in science, yet low levels of self-efficacy and self-concept in science, which might be due to the emphasis on modesty and humility in Asian cultures. How could this (i.e., high level of performance in science vs. low level of self-efficacy/self-concept among Asian students) have moderated the relationship between attitude toward science and performance in science is not clear, and warrants attention in future research.

### Type of Attitude

Our results revealed that the science self-efficacy was more strongly connected to science achievement than that of interest, societal relevance of attitude toward science, and mixed attitude. This finding was in accordance with the proposition that the effect of self-efficacy on students' learning performance could be stronger than some other dimensions (e.g., value, interest; Lam and Lau, 2014), and self-efficacy could significantly predict science learning (e.g., Kaya and Bozdag, 2016; Juan et al., 2018; Kirbulut and Uzuntiryaki-Kondakci, 2019). A meta-analysis conducted by Sun et al. (2021) also showed that there was a positive correlation between students' self-efficacy and writing achievement in a second language. That's not surprising because the personal beliefs of competence might influence their behavior and decisions (Bandura, 1993). Individuals with a high level of self-efficacy are likely to put forth more mental effort to solve problems and persist in the face of difficulties (Pajares, 1996). One notable observation in this analysis was that the association between societal relevance of attitude toward science and science achievement was notably the weakest, which was in accord with recent studies (Wang and Liou, 2017; Cohen and Chang, 2020).

### Measures of Achievement

Standardized test and unstandardized assessment for measuring science achievement could significantly affect the relationship between science attitude and science achievement. In general, the effect sizes based on unstandardized assessment were descriptively higher than those based on standardized test. This finding is in accordance with that of Mason et al. (2013) who also found that the association between self-concept or self-efficacy and science achievement measured with unstandardized test was stronger than with standardized test in fifth, eighth, and eleventh grades. One possible explanation could be that students' attitude toward science could be based on their performance in the process of science learning more than on their actual science competence measured by standardized tests (Jansen et al., 2014). Future research could comprehensively examine the association between attitude toward science and science achievement with standardized and unstandardized measures.

### Publication Year

No significant effect of publication year was found in this meta-analysis, suggesting that the association between attitude toward science and academic achievement in science does not seem to be related to the time when the studies were published. This result is consistent with that of Willson (1983) who showed that the strength of this correlation did not vary significantly over time. Despite the recent trend of putting more emphasis on STEM education, it appears that the strength of the relationship between attitude toward science and science achievement has been stable, as shown in both previous (Willson, 1983) and the present meta-analyses.

## Limitations and Future Directions

This meta-analysis shows that there is a statistically significant and robust positive relationship between students' attitude toward science and their academic achievement in science, and the strength of this association is stronger in self-efficacy than in interest, societal relevance, mixed attitude, which offers valuable insights into the intervention of students' science attitude. The study has some limitations that should be noted. First, we did not examine how gender groups could be different in the relationship between science attitude and performance in science learning, due to the very small number of studies that had such relevant information. But gender difference, or lack thereof, in science learning and in attitude toward science of students is an unsettled issue. While some studies suggested that males were more positive about science and had better academic outcomes in science than females (Jones et al., 2000; Louis and Mistele, 2012; Oluwatelure, 2015), some other studies indicated that gender showed no significant role in this context (Miller et al., 2002; Dhindsa and Chung, 2003; Oludipe, 2012). Further research is needed on how gender groups may or may not differ on these related issues.

Second, with regard to attitude toward science and science learning achievement, too few studies involved elementary school students. More specifically, only 6 of 37 studies involving elementary school students were found and included in our meta-analysis. Currently, there is growing evidence that science learning at younger age (e.g., elementary school) could be beneficial in a long term (Morgan et al., 2016; Curran and Kitchin, 2019). For example, Curran and Kitchin (2019) suggested that time spent on science instruction at younger age (kindergarten, first to third grades) could positively predict later science achievement. As a result, it should be highly beneficial to conduct relevant research involving primary school students.

Another limitation to this study was the limited set of study features that we examined as potential moderator variables (i.e., publication year, publication type, grade, geographical region, type of attitude, measures of achievement). There could be other factors (e.g., teaching experience of teachers, sampling method) that could affect the attitude toward science and science learning (Mohammadpour, 2012; Ulutan and Aktan, 2019). Future research on attitude toward science and science learning achievement may consider such and other relevant variables that may influence the constructs under study. In addition, the coded information of moderator variables also needs to be carefully considered.

Finally, the concept of science is broad, and the studies included in this meta-analysis were concerned about the overall attitude in science, but not about specific domains under science (e.g., physics, chemistry, biology, etc.). Nissen (2019) showed that female high school students had lower level of self-efficacy in physics course than in other science courses, and their level of self-efficacy in physics course was substantially lower than that of their male counterparts. Hence, it should be meaningful in future research to examine this issue (i.e., relationship between attitude and achievement) in different science subjects.

## Data Availability Statement

The original contributions presented in the study are included in the article/Supplementary Material, further inquiries can be directed to the corresponding author/s.

## Author Contributions

ZC led the study design, data collection, statistical analysis, and drafted the manuscript. PM helped to analyze the data and draft the manuscript. XC and JH helped to design the study and collect data. XF helped to draft the manuscript. All authors read and approved the final manuscript.

## Funding

This research was supported by the National Natural Science Foundation of China (Grant No. 62107018), Grant CCNU20QN025 from the Fundamental Research Funds for the Central Universities, and CCNU19TD019 from the self-determined research funds of CCNU from the colleges' basic research and operation of MOE.

## Conflict of Interest

The authors declare that the research was conducted in the absence of any commercial or financial relationships that could be construed as a potential conflict of interest.

## Publisher's Note

All claims expressed in this article are solely those of the authors and do not necessarily represent those of their affiliated organizations, or those of the publisher, the editors and the reviewers. Any product that may be evaluated in this article, or claim that may be made by its manufacturer, is not guaranteed or endorsed by the publisher.
